# Endophytic mycorrhizal fungi strengthen *Lactuca sativa* defense against *Alternaria alternata* as a sustainable biocontrol approach

**DOI:** 10.1186/s12870-026-08877-0

**Published:** 2026-05-14

**Authors:** Rabab A. Metwally, Rania S. Shehata, Reda E. Abdelhameed

**Affiliations:** 1 Botany and Microbiology Department, Faculty of Science, Zagazig University, Zagazig, 44519 Egypt; 2 Biology Department, College of science, Jazan University, Jazan, 45142 Saudi Arabia

**Keywords:** Mycorrhizal symbiosis, *Alternaria alternata*, Disease severity, Phosphatases, Polyphenolics, Plant protection, Antioxidant enzymes, Leaf spot disease

## Abstract

Most terrestrial plants can establish symbiotic relationships with arbuscular mycorrhizal (AM) fungi, which increase the host plants’ resilience to pathogens. The effect of pre-inoculation with AM fungi as a bio-agent on lettuce (*Lactuca sativa* L.) plant resistance against *Alternaria alternata* RaSh3 leaf spot disease was investigated. The findings demonstrated that in *A. alternata*-infected plants, AM fungi could effectively colonize lettuce roots at a higher rate (100%) than in non-infected plants (91.66%). According to the disease assessment, lettuce plants pre-inoculated with AM and infected with *A. alternata* RaSh3 showed a 33.33 and 30.00% reduction in disease incidence and severity, respectively. During *A. alternata* RaSh3 infection, the primary growth responses, pigment fraction, proline, and carbohydrates of lettuce plants were reduced, accompanied by increases in oxidative stress markers [malondialdehyde (87%) and hydrogen peroxide (30.8%)]. Contrarily, AM-inoculated plants showed a significant increase in growth, photosynthetic pigments, osmolytes and enzymatic and non-enzymatic antioxidant enzymes either in *A. alternata* RaSh3-infected or non-infected ones. Overall, our results highlight the significance of AM fungi in alleviating infection symptoms by increasing proline (13%), flavonoids (28.3%), and phenolic compounds (44.7%). Moreover, a boost in the enzymatic status (phosphatases, antioxidants, and phenylalanine ammonia-lyase) was detected in *A. alternata* RaSh3-infected plants due to AM inoculation, proving the essential role of its inoculation in increasing plant resistance against *A. alternata* RaSh3. Finally, this experiment has proved the sustainable defense strategy of mycorrhizal symbiosis as a new bio-agent for the biological control of *A. alternata* in lettuce plants.

## Background

The leafy vegetable crop lettuce (*Lactuca sativa* L.) is a seasonal plant and a member of the Asteraceae family and was domesticated in Egypt approximately 4,500 BC. It is a significant food and used as a fresh vegetable in salads and embraces flavonoids, carotenoids, vitamin E, antioxidants, and phenolic compounds [1–3]. The range and severity of phytopathogenic fungi, such as *Alternaria*, on a variety of crops [4–7] are on the rise globally. The genus *Alternaria* can inhabit various ecological niches and function as pathogens, endophytes, and saprotrophs [8–10]. *Alternaria* spp. affects all parts of plants, such as leaves, stems, and fruits. It is best known for causing crops to rot while they are being stored [11].

According to Bradley et al. [12], the most significant and common issues affecting lettuce were 16 fungal diseases; its production is now threatened by a fungal leaf spot disease caused by *Alternaria* spp [3, 8]. A wide range of other hosts, including sunflowers, wild and cultivated rocket, spinach, chilli peppers, *Digitalis purpurea*, basil, and cabbage, are susceptible to severe economic harm from *Alternaria* leaf spot, which can result in yield losses of up to 80% [8, 13, 14]. Necrotic lesions on leaves that resemble huge brown or black spots with distinctive concentric zonation and are frequently encircled by yellow chlorotic tissue are typical signs of diseases caused by *Alternaria* spp. The diffusion of fungal secondary metabolites that are harmful to the host causes the formation of this zone [15].

*A. alternata*, the causative agent of leaf spot disease, has been controlled using a variety of techniques, such as chemical, biological, cross-protection, agricultural practices, and resistant cultivars [3, 16–19]. The traditional method of controlling *Alternaria* leaf spot, which involves spraying synthetic chemical fungicides, has been suggested and occasionally shown positive outcomes; however, its use primarily results in detrimental side risks such as harm to human health, pollution of the environment, and a decline in effectiveness as a result of pathogen resistance to fungicides [20, 21]. Therefore, reducing the usage of chemical inputs is becoming a major priority in the pursuit of more sustainable agriculture. Therefore, to manage *Alternaria* leaf spot disease, alternatives to conventional fungicides are being looked for.

Biological control is an environmentally effective means of decreasing the dangerous effect of synthetic pesticides due to their professed increased level of safety and minimal environmental influences [8, 22, 23]. Many fungal plant diseases are thought to be well controlled by biological control, and research into novel biological agents as possible antagonists of biological control is growing [24, 25]. Furthermore, biocontrol techniques for *Alternaria* leaf spot can offer less harmful substitutes for synthetic chemical fungicides.

One of these biological techniques is mycorrhizal fungi that coexist symbiotically with plants at the root level of the majority of terrestrial plants [26]. Arbuscular mycorrhizal (AM) fungi’s inadequate exo-enzymatic apparatus prevents them from mineralizing soil organic materials [27]. Therefore, they must build a highly specialized interface with the roots of most vascular plants and establish compatible mutualistic symbiosis with host plants (obligate biotrophs) to complete their growth cycle [28, 29]. Through the systemic induction of resistance mechanisms, they are known to increase plant defense and tolerance to a variety of biotic and abiotic stimuli [30, 31]. According to Comby et al. [32] and Wang and Chen [33], by triggering several biochemical and molecular processes, mycorrhizal symbiosis can defend plants against a variety of plant diseases, including bacteria, viruses, fungi, phytoplasma, and pests. For example, inoculation with the AM fungus *Glomus etunicatum* BEG168 increased plant resistance to the causative agent of cucumber wilt disease, *F. oxysporum* f. sp. *cuncumerinum* [34]. First, enhanced plant nutrition and better photosynthesis can aid mycorrhizal plants in fending off pathogen attacks [35]. Second, by competing with below-ground pathogenic microorganisms both geographically and nutritionally, the extra-radical mycelium of AM fungi modifies the microbiota surrounding the roots [36]. In competition with harmful belowground microorganisms, the mycorrhizal extra-radical mycelium also promotes the activity of beneficial microorganisms like phosphate-solubilizing and nitrogen-fixing bacteria [37–38].

Most research focuses on how AM fungi protect plants from soil-borne illnesses [39, 40]. Nevertheless, little is known about how AM fungi, which primarily occur in the subterranean portions of plant hosts, affect the pathogens that cause diseases in the aboveground portions of plants. So, there is a dearth of information regarding how well AM inoculum works to prevent aerial plant diseases [32]. The applicability of AM fungi as a biocontrol agent against foliar diseases needs to be investigated more thoroughly. Nowadays, AM inoculums are being utilized more and more as bio-stimulants to enhance plant uptake of minerals and nutrients. Therefore, the present investigation attempts to investigate the potential of AM fungi as biostimulants and/or biocontrol agents. Therefore, the objectives of our study will emphasize the efficacy of AM fungal treatment to control *Alternaria* leaf spot caused by *Alternaria alternata* RaSh3 in *Lactuca sativa* plants, as well as the obstacles that still need to be addressed.

## Materials and methods

### Fungal materials

#### Leaf spot pathogen (*Alternaria alternata* RaSh3)

*A. alternata* RaSh3 was previously isolated from diseased pepper plant leaf samples that exhibited leaf spot disease symptoms and were gathered from several El-Sharkia governorate locations in Egypt. *A. alternata* RaSh3 was molecularly identified and deposited into the GenBank database with the accession number OK053809.1. It was previously tested and confirmed to cause leaf spot disease in *Capsicum annuum* L [8]. and *L. sativa* L [10].

*A. alternata* RaSh3 inoculum was prepared from cultures grown on potato dextrose agar (PDA) medium and incubated at 25 °C for 7 days. The developed cultures were flooded using 50 mL of sterilized water. The growth (mycelial mats and spores) was carefully scraped from the medium surface. The mycelium and obtained spore suspension were filtered through sterilized cheesecloth to eliminate mycelial fragments, and the fungal suspension was adjusted (10^7^ cfu/mL) and was used for spraying leaves in vivo.

#### AM fungal inoculum

The used AM fungal spores were *Funneliformis constrictum*, *F. mosseae*, *Gigaspora margarita*, and *Rhizophagus irregularis*. These spores were originally isolated from rhizospheric soils of various plant species by the wet sieving technique [41]. The inoculum was cultured for six months for two cycles on the roots of Sudan grass (*Sorghum sudanenses* Pers.), a suitable trap plant, in a sterilized substrate of a 1:1 mixture of sand and clay, where the colonization index was 100%. The AM fungal inoculum enclosed hyphae, soil, colonized root pieces, and spores.

### In vivo challenge of AM fungal effect against *A. alternata* RaSh3 leaf spot in lettuce plants

#### Experimental design and growth condition

The impact of AM fungal inoculation on the biocontrol of *A. alternata* RaSh3 and lettuce plant growth was assessed in a pot experiment conducted under a controlled environment in the greenhouse of the Botany and Microbiology Department, Faculty of Science, Zagazig University (temperature: 25 °C; relative humidity: 70%; photoperiod: 13 light/11 dark h). Lettuce (*Lactuca sativa* L.) (Lettuce Nader, US, GP 95%) seeds were germinated in slotted polystyrene seedling trays filled with peat moss in a nursery greenhouse for seedling development. Two-week-old lettuce seedlings were transplanted into sterilized plastic pots (24 cm in diameter and 30 cm in height) with 3 kg of sterilized soil. The pots at first were divided into 2 groups (AM and non-AM inoculated). The AM-inoculum was added as 50 g/pot at the sowing stage. The experiment included 20 pots (five replicates of 4 treatments), as follows: control, AM-inoculated, *A. alternata-*infected, and AM + *A. alternata-*infected. The *A. alternata* RaSh3 was applied on the surface of healthy plant leaves two weeks after transplantation by pipetting droplets of *A. alternata* RaSh3 suspension onto the leaves. The infected plants were covered with plastic bags for 24 h to maintain high humidity levels to ensure the infection occurred. For the control and AM treatments, plants were just sprayed with tap water. The appearance of disease symptoms was noted three weeks after pathogen application. *A. alternata* RaSh3 was re-isolated from diseased leaves of lettuce, and its identity was confirmed.

#### Leaf spot disease assessment

Visual observation was used for assessing the leaf spot disease symptoms on lettuce leaves. The disease incidence (DI) of the infected plants three weeks after pathogen application was calculated according to the following formula:

$$\mathrm{DI} (\%) = \frac{\text{Number of infected plants}}{\text{Total number of plants}} \times 100$$

Moreover, ten plants from each treatment were evaluated for disease severity (DS) using a 0–5 scale based on the percentage of leaf area covered by necrotic lesions [42]. After observing lettuce plants, these scale values were then converted into DS percentage values with the help of the Townsend and Heuberger [43] formula. Also, the percentage of disease reduction (DR) was determined based on DI results [44]. DS and DR were calculated according to the following formulas below:

$$\mathrm{DS} (\%) =\left[ \frac{\sum (Scale value \times \text{the number of plant leaves evaluated in the scale)}}{\text{(Max.disease rating} \times \text{Total number of observed plant leaves}}\right] \times 100$$

$$\mathrm{DR} (\%) = \frac{\text{DI of positive control - DI of treated with AM}}{\text{DI of positive control}} \times 100$$

#### Mycorrhizal features

Root colonization was checked after 5 weeks of AM fungal inoculation by cleaning fresh lettuce roots and cutting them into small pieces (1 cm in length). Excised root segments were then heated at 90 °C in 10% potassium hydroxide for 10 min and then acidified with 1 N hydrochloric acid (HCl) for 3 min and finally stained with trypan blue (Sigma, St. Louis, MO, USA) in lactophenol (0.05%) at 90 °C for 7 min. The excess stain was removed with 50% lactophenol. Randomly selected root segments were visualized at 10x magnification using a light microscope (Leitz WETZLAR, Wetzlar, Germany). The mycorrhizal formation was checked following the MYCOCALC program (http://www2.dijon.inra.fr/mychintec/Mycocalc-prg/download.html). Specifically, the following criteria were taken into account: M%, the intensity of mycorrhizal colonization in each examined root segment; F%, the proportion of colonized root fragments in the sample under analysis; and A%, the arbuscular abundance in colonized root fragments.

#### Phenotypic traits

The lettuce plants were employed to evaluate the impact of AM fungal inoculation and *A. alternata* RaSh3 infection on various growth parameters, including shoot height (SH), root length (RL), shoot (SFwt) and root fresh weight (RFwt) (g), and shoot (SDwt) and root dry weight (RDwt) (g). Both SDwt and RDwt were taken by drying roots and shoots in a hot air oven (48 h, 80 °C). Moreover, leaf length, width, Fwt, and Dwt were measured. Carleton and Foote’s [45] protocol was used for the measurement of leaf area (cm^2^) using the following formula:

$$\mathrm{L}\mathrm{e}\mathrm{a}\mathrm{f}\:\mathrm{a}\mathrm{r}\mathrm{e}\mathrm{a}\:\left({\mathrm{c}\mathrm{m}}^{2}\right)=\:\mathrm{m}\mathrm{a}\mathrm{x}\mathrm{i}\mathrm{m}\mathrm{u}\mathrm{m}\:\mathrm{l}\mathrm{e}\mathrm{a}\mathrm{f}\:\mathrm{l}\mathrm{e}\mathrm{n}\mathrm{g}\mathrm{t}\mathrm{h}\:\times\:\:\mathrm{m}\mathrm{a}\mathrm{x}\mathrm{i}\mathrm{m}\mathrm{u}\mathrm{m}\:\mathrm{l}\mathrm{e}\mathrm{a}\mathrm{f}\:\mathrm{w}\mathrm{i}\mathrm{d}\mathrm{t}\mathrm{h}\:\times\:\:0.75$$

Where 0.75 is the correction factor.

The mycorrhizal dependency (MD) of each treatment was calculated according to the ratio of Dwt of plants with and without AM fungi [46] according to the following equation:

$$\mathrm{MD} (\%)= \frac{\text{Dwt.of AM plants - Dwt of non.AM plants}}{\text{Dwt.of AM plants}} \times 100$$

#### Photosynthetic pigment content

It was measured by putting 1 g of lettuce plant leaf with 10 mL of acetone (80%) in dark bottles, followed by incubation for 24 h in the dark. Chl a and Chl b were calculated by measuring the optical density (OD) at 663, 644, and 452.5 nm [47] using a UV-visible spectrophotometer, RIGOL (Model Ultra-3660).

$$\:\mathrm{C}\mathrm{h}\mathrm{l}\:\mathrm{a}\:({\upmu}\mathrm{g}/\mathrm{m}\mathrm{g}\:\mathrm{F}\mathrm{w}\mathrm{t})=({12.72}_{A663}-{2.69}_{A644})\times\:\mathrm{V}/(\mathrm{m}\times\:1000)$$

$$\mathrm{C}\mathrm{h}\mathrm{l}\:\mathrm{b}\:({\upmu}\mathrm{g}/\mathrm{m}\mathrm{g}\:\mathrm{F}\mathrm{w}\mathrm{t})=({22.9}_{A644}-{4.68}_{A663})\times\:\mathrm{V}/(\mathrm{m}\times\:1000)$$

$$\mathrm{T}\mathrm{o}\mathrm{t}\mathrm{a}\mathrm{l}\:\mathrm{C}\mathrm{h}\mathrm{l}\:({\upmu}\mathrm{g}/\mathrm{m}\mathrm{g}\:\mathrm{F}\mathrm{w}\mathrm{t})=\mathrm{C}\mathrm{h}\mathrm{l}\:\mathrm{a}\:+\:\mathrm{C}\mathrm{h}\mathrm{l}\:\mathrm{b}$$

$$\mathrm{C}\mathrm{a}\mathrm{r}\mathrm{o}\mathrm{t}\mathrm{e}\mathrm{n}\mathrm{o}\mathrm{i}\mathrm{d}\mathrm{s}\:\left({\upmu}\mathrm{g}/\mathrm{m}\mathrm{g}\mathrm{F}\mathrm{w}\mathrm{t}\right)=\left({4.2}_{A452.5}\right)-\left(0.0264\:\mathrm{C}\mathrm{h}\mathrm{l}.\:\mathrm{a}+0.426\:\mathrm{C}\mathrm{h}\mathrm{l}.\:\mathrm{b}\right)\times\:\mathrm{V}/(\mathrm{m}\times\:1000)$$

#### Biochemical traits

##### Malondialdehyde assay (MDA) and hydrogen peroxide (H_2_O_2_) level

MDA was measured by taking 0.5 g of lyophilized lettuce leaves in 5 mL of 0.1% trichloroacetic acid (TCA). The resulting mixture was then centrifuged (12,000 rpm, 20 min). The supernatant was mixed with 2 mL of reaction buffer containing 20% TCA and 0.5% thiobarbituric acid (TBA) and kept for incubation at 98 °C for 15 min. Again, centrifugation was performed for 10 min. MDA activity was checked (600 and 532 nm) [48]. Hydrogen peroxide (H_2_O_2_) level (µg/mg Fwt) was quantified at 390 nm [49]. The supernatant (0.5 mL) was added to 1 M potassium iodide (2 mL) besides 0.5 mL of 10 mM potassium phosphate buffer (pH 7.0).

##### Lettuce osmo-protective compounds

The protein content in fresh lettuce leaves was determined by mixing 1 g of leaf sample in 0.05 M potassium phosphate buffer (pH: 7). The resulting solution was then centrifuged (12,000 rpm, 4 °C, 10 min). In total, 0.5 mL of extracted sample was mixed in 2.5 mL of freshly prepared alkaline copper reagent, followed by incubation for 10 min. The OD was checked at 700 nm after adding 0.5 mL Folin-Ciocalteau reagent for 30 min [50]. Total soluble carbohydrates were determined in dried lettuce shoot tissues using phenol-sulfuric acid reagent [51] by adding 100 µL of HCl-plant extract (2.5 N) to 5 mL of H_2_SO_4_, followed by 1 mL of phenol (5%). The OD of the sample was measured at 490 nm. A standard glucose curve was plotted using a glucose range (20–400 µg/mL) to determine total soluble carbohydrates in terms of µg/mL and was then expressed as µg/mg Dwt. The proline content was quantified from aliquots of a known lettuce Fwt using the method outlined by Bates et al. [52] after homogenizing in 5 mL of 3% aqueous sulfosalicylic acid, and its concentration was computed as follows:

$$\mathrm{P}\mathrm{r}\mathrm{o}\mathrm{l}\mathrm{i}\mathrm{n}\mathrm{e}\:\mathrm{c}\mathrm{o}\mathrm{n}\mathrm{c}\mathrm{e}\mathrm{n}\mathrm{t}\mathrm{r}\mathrm{a}\mathrm{t}\mathrm{i}\mathrm{o}\mathrm{n}\:\left({\upmu}\mathrm{g}/\mathrm{g}\mathrm{F}\mathrm{w}\mathrm{t}\right)=\frac{\left({\upmu}\mathrm{g}\:\mathrm{p}\mathrm{r}\mathrm{o}\mathrm{l}\mathrm{i}\mathrm{n}\mathrm{e}/\mathrm{m}\mathrm{L}\:\times\:\:\mathrm{m}\mathrm{L}\:\mathrm{t}\mathrm{o}\mathrm{l}\mathrm{u}\mathrm{e}\mathrm{n}\mathrm{e}\right)}{115.5\:\mathrm{x}\:\:\mathrm{g}\:\mathrm{F}\mathrm{w}\mathrm{t}\:\mathrm{o}\mathrm{f}\:\mathrm{s}\mathrm{a}\mathrm{m}\mathrm{p}\mathrm{l}\mathrm{e}}$$

##### Lettuce polyphenolic compound measurement

After grinding a known Fwt (1 g) of lettuce leaves with 95% ethanol, total phenolic and flavonoid contents were quantified. The quantity of total extracted phenolics was expressed in µg of gallic acid equivalent (GAE)/mg Fwt using gallic acid as the standard, and the absorbance was read at 650 nm [53]. The quantification of total flavonoid content in lettuce expressed as µg/mg Fwt of quercetin equivalent (QE) at 510 nm, as Shraim et al. [54] outlined, by the AlCl_3_ colorimetric technique. Moreover, TAC in methanol extracts was measured using ammonium molybdate reagent [55]. After 90 min of incubation in a boiling water bath, the tubes were cooled, and the absorbance at 695 nm was measured. The TAC was given in µg of comparable ascorbic acid per mg of Fwt.

##### Lettuce enzyme activities

Ascorbate peroxidase (APX) was determined by adopting the methodology of Nakano and Asada [56] by monitoring the oxidation of ascorbate as a substrate in the presence of H_2_O_2_. The OD of the solution was measured at 290 nm. The CAT activity in lettuce leaves was calculated following the breakdown of H_2_O_2_ for 1 min., and the decrease in absorbance was observed at 240 nm using the protocol of Aebi [57]. Peroxidase (POD) activity was estimated following Chance and Maehly’s [58] method at 470 nm. Phenylalanine ammonia lyase (PAL) activity was determined in lettuce leaves by adopting the methodology of McCallum and Walker [59]. The absorbance of the reaction mixture was measured at 290 nm (37 °C, 30 s each). The reaction mixture was made by adding 200 µL of phenylalanine (40 mM) to 200 µL of enzyme extract and 400 µL of 0.06 M borate buffer, and all were incubated for 30 min. Moreover, phosphatase (Acid [ACP] and alkaline [ALP]) enzymes were determined after macerating a known root Fwt in 0.1 M borate buffer (pH 8.5), and the homogenate was centrifuged at 6000 rpm for 10 min. The soluble phosphatase activity was estimated quantitatively in the supernatant [60]. One enzyme activity unit was defined as 1 nmol of PNP/min.

### Statistical analysis

The data presented are means ± standard error (SE). One-way ANOVA, followed by the Duncan test at the *p*-value ≤ 0.05 significance level, was carried out using the SPSS (SPSS Statistics version 15) software package for expressing the statistical significance. Two-way ANOVA was conducted to evaluate the main and interactive effects of *A. alternata* RaSh3 infection and AM fungal colonization on various lettuce-measured variables. Figures were drawn using OriginPro 2017.

## Results

### Influence of AM fungi on lettuce leaf spot disease parameters

The biocontrol efficiency of AM fungal inoculation was checked against *A. alternata* RaSh3 after three weeks of pathogen application. Table 1 shows the disease incidence (DI), severity (DS), and reduction (DR) of lettuce plants infected with *Alternaria* leaf spot disease in response to AM fungal colonization. Lettuce plants that had not been infected with *A. alternata* RaSh3 [control and AM-inoculated treatments] showed no disease signs. The solely infected (non-AM-infected) lettuce plants had the highest DS and DI, measuring 60% and 90%, respectively. On the other hand, DS [42%] and DI [60%] were significantly lower in *A. alternata*-infected and colonized with AM fungi. These results proved that AM fungal colonization decreased DS and DI with a DR of 33.33% as compared to non-mycorrhizal-infected plants.

**Table 1:** Different disease assessment parameters and mycorrhization levels in lettuce plants infected with *A. alternata* RaSh3 leaf spot disease in response to arbuscular mycorrhizal (AM) fungal colonization at 21 days post-infection

Treatments	Disease incidence (DI %)	Disease severity (DS %)	Disease reduction (DR%)	Mycorrhizal frequency (F%)	Mycorrhizal intensity (M%)	Arbuscules abundance (A%)
Control	0c	0c	0b	0c	0c	0c
*A. alternata*	90 ± 2.31a	60 ± 1.21a	0b	0c	0c	0c
AM	0c	0c	0b	91.66 ± 2.33b	74.59 ± 1.71b	50.00 ± 1.02b
AM + *A. alternata*	60 ± 1.32b	42 ± 0.97b	33.33 ± 0.52a	100 ± 0.00a	81.00 ± 1.85a	58.33 ± 1.11a

The presence of different letters next to the data values in each column indicates that the differences were significant at *p*-value ≤ 0.05

### Influence of *A. alternata* RaSh3 on lettuce root colonization

**Fig. 1:**
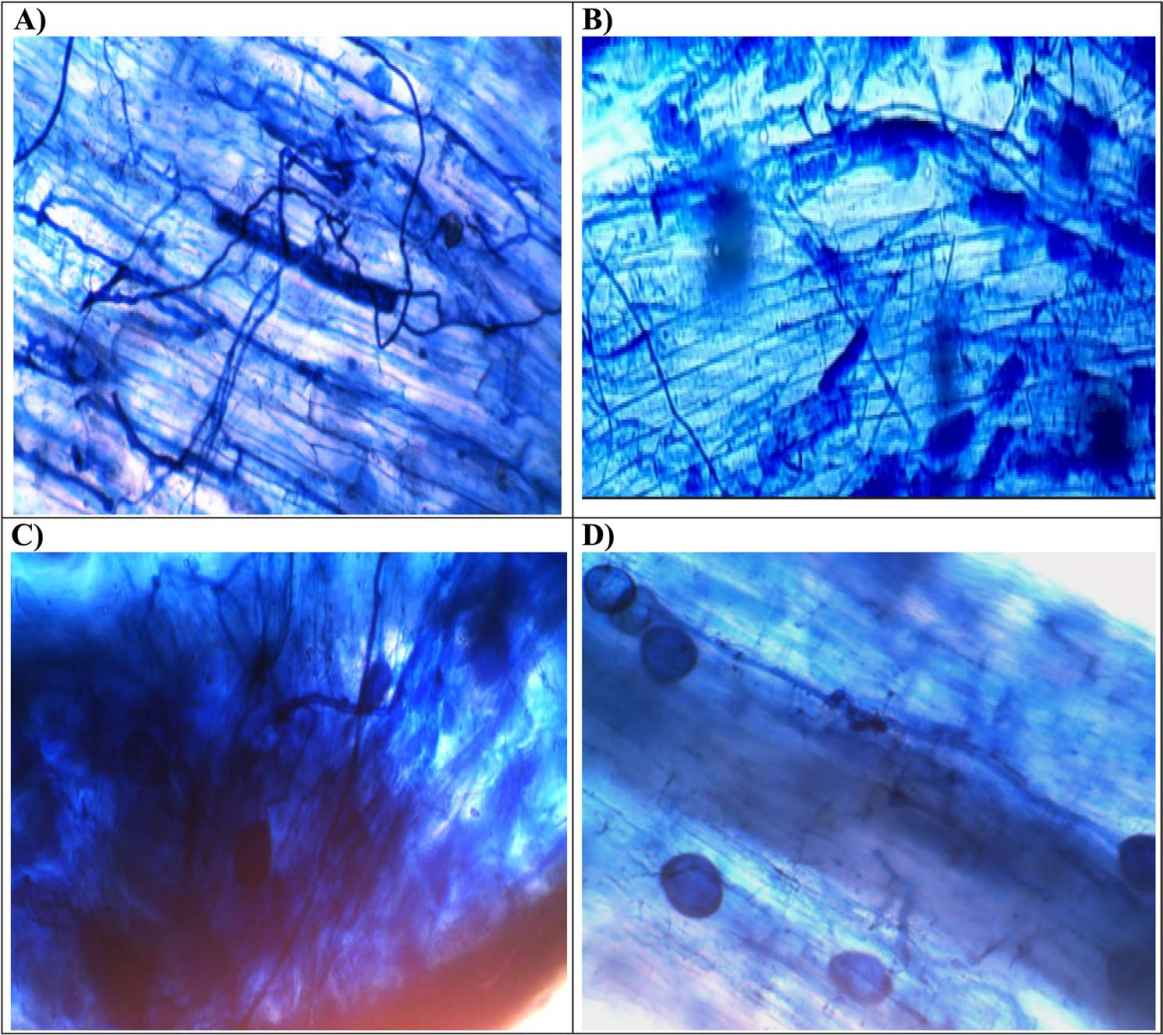
Mycorrhizal structures observed in lettuce root segments (10x), 35 days after AM fungal inoculation (trypan blue staining). **A** Inoculated root segments with AM fungal hyphae, (**B**) Inoculated roots with arbuscules and hyphae, **C** and **D**) Inoculated roots with AM fungal vesicles and hyphae

After thirty-five days of mycorrhizal application, the AM fungal colonization rates of lettuce plant roots were quantified by light microscopic analysis following trypan blue staining (Fig. 1). Table 1 shows the mycorrhizal colonization rates in the control and infected roots of lettuce exhibiting *Alternaria* leaf spot. According to the results, non-AM colonized lettuce roots showed no signs of mycorrhization. Contrariwise, the other treatments with AM fungal inoculum displayed colonization to varied degrees. Through microscopic analysis, several prominent mycorrhizal structures were seen in the lettuce root segments (Fig. 1A-D). The AM-inoculated roots displayed the typical mycorrhizal fungal structures, including fungal hyphae (Fig. 1A), arbuscules, a special structure of AM fungi that develops inside the root cells where nutrient exchange between the plant and fungus occurs (Fig. 1B), and vesicles, which are lipid storage structures produced in fully developed mycorrhizas (Fig. 1C and D).

According to the data, AM-colonized and infected lettuce plants exhibited the highest levels of arbuscule frequency (58.33%), colonization intensity (81%), and colonization frequency (100%). These findings demonstrated that our AM inoculum could effectively colonize *A. alternata* RaSh3-infected or non-infected lettuce roots and that the colonization rose in plants dually treated with *A. alternata* RaSh3 and AM fungi. Overall, these suggest that the intraradical development of AM fungi is not significantly impacted when lettuce is infected with leaf spot disease.

#### Growth response of lettuce upon AM application and *A. alternata* RaSh3 infection

**Fig. 2:**
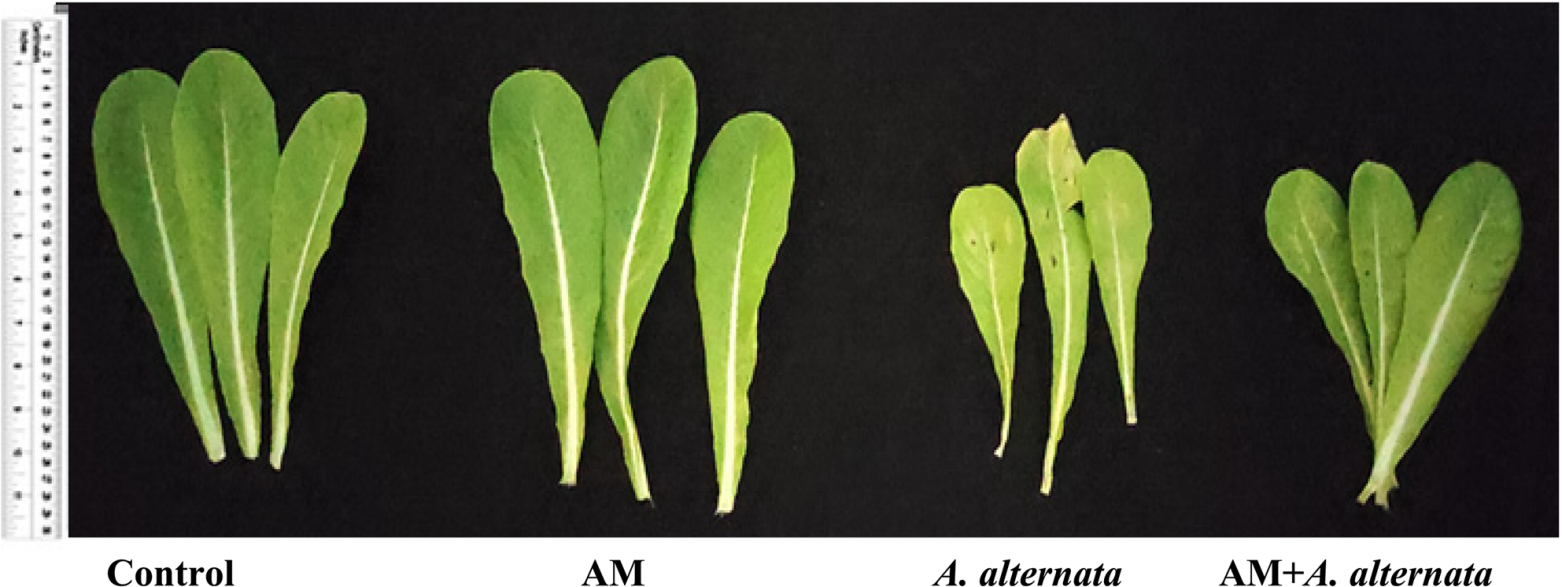
Effect of arbuscular mycorrhizal (AM) colonization and *A. alternata* RaSh3 pathogen infection on the morphological appearance of lettuce leaves

The phenotypic traits of lettuce plants were assessed and showed that plants afflicted with *A. alternata* RaSh3 had considerably lower values of all the measured growth parameters (length, Fwts, and Dwts of shoot, root, and leaf) as compared to the healthy controls (Fig. 2 and Table 2). Also, visual observation of AM-inoculated plants and comparison with the control and the *A. alternata* RaSh3-infected plants showed a significant enhancement in lettuce growth promotion under control or *A. alternata* RaSh3 stress conditions (Fig. 2). As well, the other leaf parameters (number, width, and area) were reduced in *A. alternata* RaSh3-treated lettuce plants. When compared to *A. alternata* RaSh3-infected lettuce plants, mycorrhizal inoculation markedly increased SH and RL (37.5 and 31.7%); SFwt and RFwt (20.8 and 39.8%); SDwt and RDwt (46.3 and 79.7%); and leaf width and area (16.1 and 24.8%).

**Table 2:** Mean amounts (± standard error) of shoot, root, and leaf parameters of lettuce plants exposed to arbuscular mycorrhizal (AM) colonization and *A. alternata *RaSh3 pathogen infection

Treatments		Control	*A. alternata*	AM	AM+*A. alternata*
Parameters					
Shoot	Height (SH; cm)	10.00±0.264c	8.00±0.212d	13.50±0.357a	11.00±0.291b
	SFwt (g)	12.08±0.319b	9.22±0.244c	15.01±0.397a	11.14±0.294b
	SDwt (g)	0.993±0.026b	0.668±0.018c	1.082±0.028a	0.977±0.026b
Root	Length (RL; cm)	8±0.211b	6±0.158c	9.5±0.251a	7.9±0.209b
	RFwt (g)	0.95±0.025c	0.93±0.024c	1.45±0.038a	1.3±0.034b
	RDwt (g)	0.105±0.0028b	0.1035±0.0027b	0.1928±0.0051a	0.186±0.0049a
Leaf	Length (cm)	16.1±0.426a	12.75±0.337b	16.85±0.445a	13.7±0.362b
	Fwt (g)	1.12±0.029b	0.57±0.015d	1.57±0.042a	0.93±0.024c
	Dwt (g)	0.0843±0.002b	0.0391±0.001d	0.1162±0.003a	0.0704±0.0018c
	Number (No.)	10.33±0.273b	9±0.238c	12±0.317a	10±0.365b
	Width (cm)	5.6±0.148a	4.35±0.115c	6.05±0.16a	5.05±0.1336b
	Area (cm^2^)	70.77±1.87b	43.53±1.15d	80.024±2.11a	54.31±1.44c

Duncan test was carried out to compare means. The presence of different letters next to the data values in each row indicates that the differences were significant at *p-*value ≤ 0.05

*Fwt* Fresh weight, *Dwt* Dry weight

Duncan test was carried out to compare means. The presence of different letters next to the data values in each row indicates that the differences were significant at *p*-value ≤ 0.05. Fwt: fresh weight; Dwt: dry weight.

### Physio-biochemical responses of lettuce upon AM application and *A. alternata* RaSh3 infection

#### Effect on photosynthetic pigments

The contents of photosynthetic pigments are closely related to the plant photosynthetic capacity; this study investigated the Chl and carotenoid contents of lettuce leaves under different treatments. Their contents decreased by *A. alternata* RaSh3 but were considerably reinstated by AM inoculation (Table 3). The maximum decrease in Chl a (20.8%), Chl b (24.6), Chl (a + b) (22.8%), and carotenoid (23.5%) contents was reported in pathogen-challenged lettuce plant samples compared to control plants, where the necrotic spots produced on pathogen-infected leaves were more prominent, indicating that the pathogen induced malfunctioning of photosynthetic machinery (Fig. 2). Most obviously, the photosynthetic pigments in the mycorrhizal plants were greater than those of the non-mycorrhizal plants, whether the plants were infected or not, so mycorrhizal colonization reduced the negative effects of the pathogen; it restored photosynthetic pigments (Chl a, Chl b, Chl (a + b), and carotenoid contents by 12.2%, 25.5%, 18.8%, and 19.3%, respectively) relative to non-inoculated and infected ones.

**Table 3:** Mean amounts (± standard error) of photosynthetic pigments of lettuce leaves exposed to arbuscular mycorrhizal (AM) colonization and *A. alternata* RaSh3 pathogen infection

Treatments		Control	*A. alternata*	AM	AM + *A. alternata*
Parameters					
Chlorophyll (µg mg^− 1^ Fwt)	Chl a	0.732 ± 0.019ab	0.552 ± 0.015c	0.805 ± 0.021a	0.693 ± 0.018b
	Chl b	0.683 ± 0.018b	0.541 ± 0.014d	0.784 ± 0.021a	0.607 ± 0.016c
	Total (a + b)	1.415 ± 0.037b	1.093 ± 0.028c	1.589 ± 0.042a	1.299 ± 0.034b
Carotenoids (µg mg^− 1^ Fwt)		0.631 ± 0.016a	0.483 ± 0.013c	0.650 ± 0.017a	0.576 ± 0.015b
Total pigments (µg mg^− 1^ Fwt)		2.045 ± 0.054b	1.576 ± 0.042d	2.239 ± 0.059a	1.876 ± 0.049c

Duncan test was carried out to compare means. The presence of different letters next to the data values in each row indicates that the differences were significant at *p*-value ≤ 0.05

#### Effect on MDA and H_2_O_2_ generation

**Fig. 3:**
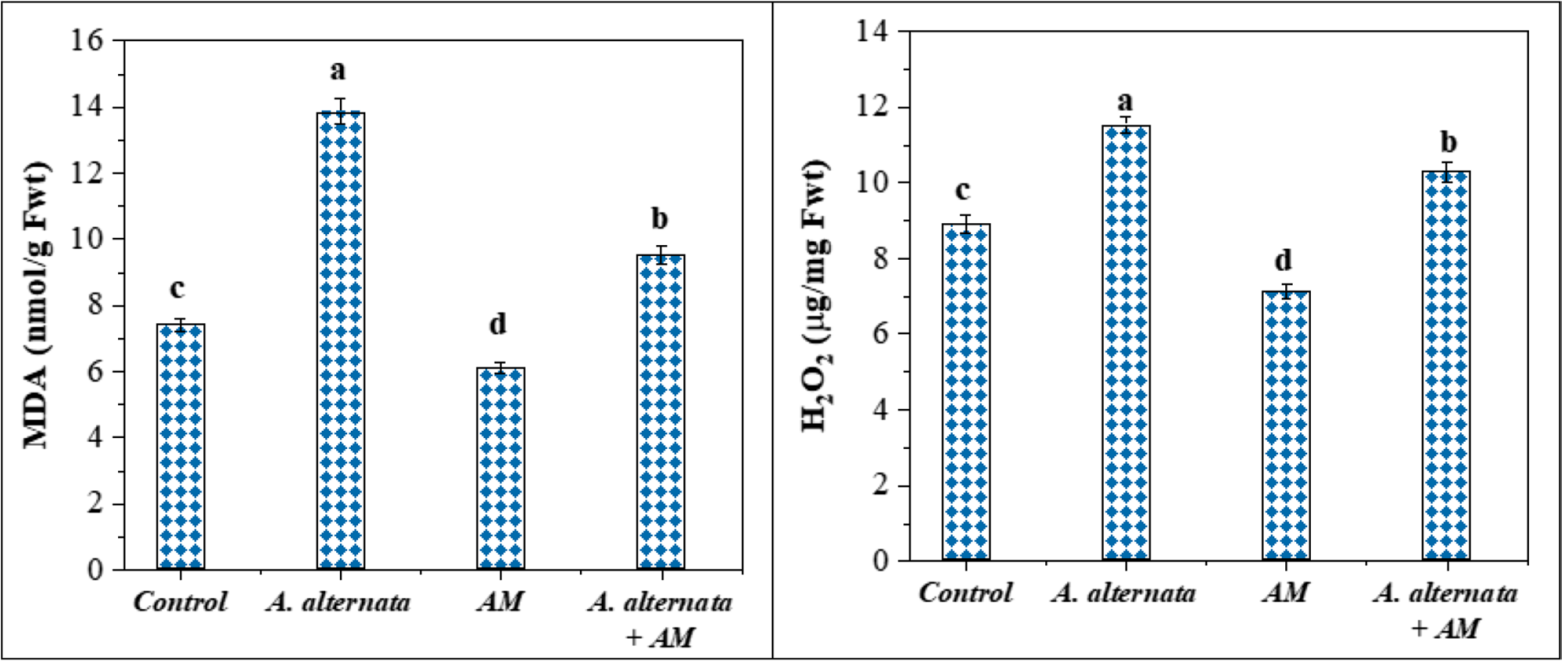
Effect of arbuscular mycorrhizal (AM) fungal and *A. alternata* RaSh3 infection on lettuce stress biomarkers (malondialdehyde: MDA and hydrogen peroxide: H_2_O_2_). *The values are the means of 5 replicates ± standard error. The same letter above each column indicates no significant difference between the treatments (*p* -value *≤* 0.05) as determined by Duncan test

Data analysis for MDA and H_2_O_2_ levels showed a highly significant difference in lettuce plants under *A. alternata* RaSh3 infection and mycorrhizal inoculation. According to our findings (Fig. 3), *A. alternata*-induced infected spots on lettuce leaves resulted in a significant increase in the amount of MDA (13.87 ± 0.367a) and H_2_O_2_ (11.64 ± 0.208a). The lowest MDA (6.112 ± 0.162d) and H_2_O_2_ (7.12 ± 0.188d) contents were noticed in AM-colonized lettuce. Interestingly, compared with the infected lettuce plants, generation of MDA and H_2_O_2_ was lowered by 31.4 and 11.6% in *A. alternata*-infected plants due to mycorrhizal symbiosis.

#### Effect on osmo-protective compounds

**Fig. 4:**
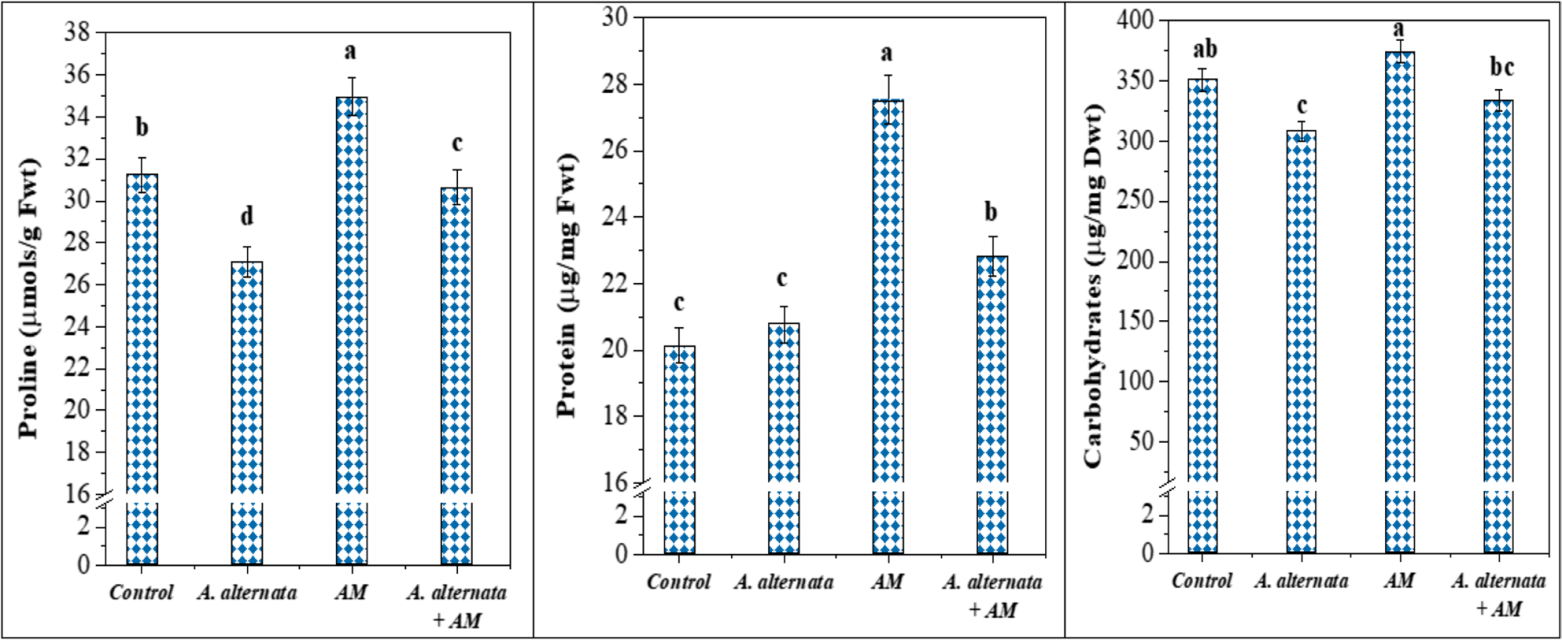
Effect of arbuscular mycorrhizal (AM) fungal colonization and *A. alternata* RaSh3 infection on lettuce osmo-protective compounds (proline, protein and carbohydrates). *The values are the means of 5 replicates ± standard error. The same letter above each column indicates no significant difference between the treatments (*p* -value *≤* 0.05) as determined by Duncan test

Maintaining cellular osmotic balance is beneficial in alleviating the negative effects of pathogen attack. A highly significant difference was detected in osmo-protective compounds (proline, protein, and carbohydrates) in lettuce leaves due to mycorrhizal colonization and *A. alternata* RaSh3 infection (Fig. 4). In *A. alternata*-infected plants, a significant decline in proline and carbohydrates was observed, while a non-significant increase in protein content was noticed relative to the control plants. Most clearly, the AM-colonized lettuce plants showed significant enhancement in proline (11.9%), protein (36.7%), and carbohydrates (6.7%). A noteworthy result is that the inoculation of infected lettuce plants with AM fungi increases proline, protein, and carbohydrate contents in their leaves by 13.1, 9.9, and 8.2%, respectively, as compared to *A. alternata-*infected plants.

#### Effect on polyphenolic compounds

**Fig. 5:**
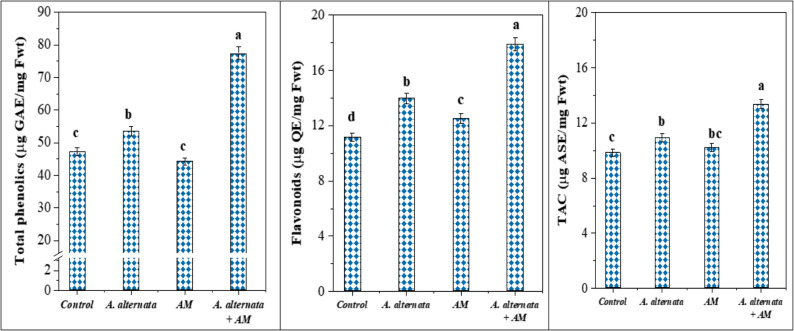
Effect of arbuscular mycorrhizal (AM) fungal colonization and *A. alternata* RaSh3 infection on lettuce non-enzymatic antioxidant (total phenolic and flavonoids) and total antioxidant capacity (TAC). *The values are the means of 5 replicates ± standard error. The same letter above each column indicates no significant difference between the treatments (*p* -value *≤* 0.05) as determined by Duncan test

Lettuce inoculation with AM fungi before *A. alternata* RaSh3 infection conferred pathogen disease tolerance and enhanced the polyphenolic compound content in their leaves. Our results showed (Fig. 5) that the pathogen-infected plants exhibited higher total phenol and flavonoid content (53.54 µg GAE/mg Fwt and 13.96 µg QE/mg Fwt) as compared with the control (47.17 µg GAE/mg Fwt and 11.17 µg QE/mg Fwt). Most importantly, the *A. alternata* infection of AM-inoculated lettuce plants revealed significant effects on phenolic and flavonoid content as recorded in two-way analysis (Table 5). Their dual application maximize their contents (77.462 and 17.91) with an increase of 64.2% and 60.3%, respectively, relative to the control (Fig. 5), which indicates the potential role of the mycorrhizal symbiosis in tolerating *A. alternata* infection.

#### Effect on antioxidant enzyme activity and total antioxidant capacity

In *A. alternata* RaSh3-infected lettuce, the activities of antioxidant enzymes, including CAT, POD, and APX, were boosted by 88.7, 8.7, and 26.5%, respectively, compared with control ones (Table 4). Consequently, the total antioxidant capacity was increased in these plants by 10.9% (Fig. 5). In addition, a non-significant stimulation of these enzymes was observed in lettuce plant leaves owing to the mycorrhizal inoculation. Meanwhile, the greatest amounts of enzyme activities were detected in *A. alternata-*infected and AM-inoculated plants as compared with the controls. The upregulation of antioxidant enzymes due to mycorrhizal inoculation protected the *A. alternata-*infected lettuce plants by imparting fast removal of ROS.

**Table 4:** Effect of arbuscular mycorrhizal (AM) colonization and *A. alternata* RaSh3 infection on lettuce enzyme activities [phenylalanine ammonia lyase (PAL), enzymatic antioxidant (ascorbate peroxidase: APX, peroxidase: POD and catalase: CAT) and phosphatases (acid phosphatase: ACP and alkaline phosphatase: ALP)]

Treatments	PAL (U/min)	APX (U/min/g Fwt)	POD (U/min/g Fwt)	CAT (U/min/g Fwt)	ACP (nmol PNP/g Fwt/min)	ALP (nmol PNP/g Fwt/min)
Control	595.41 ± 15.75c	16.657 ± 0.441c	1.892 ± 0.053b	0.159 ± 0.0042c	69.096 ± 1.83b	32.4708 ± 0.859bc
*A. alternata*	660.22 ± 16.3b	21.043 ± 0.557b	2.057 ± 0.0544b	0.300 ± 0.0079b	67.471 ± 1.78b	30.6375 ± 0.811c
AM	612.16 ± 16.19c	18.214 ± 0.482c	1.935 ± 0.051b	0.166 ± 0.0041c	82.054 ± 2.17a	38.5125 ± 1.02a
AM *+ A. alternata*	686.22 ± 18.15a	22.929 ± 0.607a	2.421 ± 0.064a	0.422 ± 0.0112a	79.263 ± 2.09a	36.8042 ± 0.921b

Data are the mean amounts (± standard error). Duncan test was carried out to compare means. The presence of different letters next to the data values in each column indicates that the differences were significant at *p*-value ≤ 0.05

#### Effect on phenylalanine ammonia-lyase (PAL) activity

The concerns of mycorrhizal colonization and *A. alternata* RaSh3 infection on PAL activity of lettuce plants are represented in Table 4. A notable rise in PAL with *A. alternata* was illustrated with an 11% increase compared with the controls. The combined treatment of mycorrhizal symbiosis and *A. alternata* showed the most rise compared with the corresponding pathogen-treated plants and untreated control.

#### Effect on the activity of phosphatases

The activity of phosphatases in lettuce roots was greatly impacted by mycorrhizal colonization and *A. alternata* RaSh3 infection (Table 4) and their interaction, as revealed in two-way ANOVA analysis (Table 5). Our results displayed that *A. alternata* RaSh3-infected plants revealed a non-significant decline in ACP and ALP activities (67.47 and 30.63 nmol PNP/g Fwt/min) as compared with the control (69.09 and 32.47 nmol PNP/g Fwt/min). Most clearly, mycorrhizal colonization promoted their activities compared with the untreated control plants. The most obvious result is more augmentation in both activities by the combined treatment of mycorrhizal symbiosis and *A. alternata* recording, an increase of 14.7% in the activity of ACP while an increase of 13.3% in the activity of ALP relative to the corresponding control.

**Table 5:** Significance levels (F-values) of arbuscular mycorrhizal (AM) colonization, *A. alternata* RaSh3 infection and their interactions on the measured variables based on two-way ANOVA analysis

Variables	AM	*A. alternata*	AM + *A. alternata*
SH	129.17***	61.91***	0.764ns
RL	65.36***	73.27***	0.905ns
SFwt	57.9***	111.5***	11.15*
RFwt	63.4***	73.9***	19.39**
SDwt	194.5***	7.42ns	4.34ns
RDwt	444.9***	0.939ns	0.359ns
Leaf length	4.621ns	67.56***	0.064ns
Leaf width	16.8**	64.3***	0.794ns
Leaf area	35.22***	246.37***	12.20*
Chl a	27.49**	54.2***	5.85*
Chl b	28.4**	93.1***	2.5ns
Chl a + b	27.97**	71.95***	0.21ns
Carotenoids	12.93**	50.8***	5.64*
Total pigments	22.84**	65.2***	1.075ns
H_2_O_2_	37.4***	134.77***	0.745ns
MDA	121.9***	369.6***	35.34***
Proline	19.47**	26.5**	0.01ns
Protein	60.4***	11.13**	19.39**
Carbohydrates	7.42*	21.28**	0.012ns
Phenolics	48.02***	172.08***	79.25***
Flavonoids	50.16***	120.24***	12.2**
CAT	63.67***	744.85***	70.25***
POD	7.56*	24.3**	14.22**
APX	10.7*	75.2***	0.099ns
TAC	22.7**	50.97***	12.3**
PAL	6.8*	8.13*	2.56ns
ACP	217.9***	8.689*	47.26***
ALP	142.1***	0.44ns	12.56**

Significance levels: *p < 0.05; **p < 0.01; ***p < 0.001

*ns* Non-significant effect

## Discussion

Although the *A. alternata* RaSh3 isolate used in this study was originally obtained from pepper, its application to *L. sativa* L. is supported by the well-documented broad host range of this pathogen. *A. alternata* is known to infect a wide variety of plant species, including leafy vegetables, and cross-host pathogenicity has been frequently reported [14, 61–64]. The ability of the RaSh3 isolate to induce characteristic leaf spot disease symptoms on lettuce plants was last conducted [10], and it was chosen to explore the potential of AM fungi to alleviate its disease symptoms in lettuce plants. According to the current findings, *A. alternata* RaSh3 caused leaf spot disease in lettuce plants. Spore germination on leaf surfaces is the first step in *A. alternata* infection, which is followed by hyphal penetration through stomata or wounds. Necrotic lesions and chlorosis are caused by the pathogen’s release of host-specific toxins and enzymes that break down cell walls, such as cellulases and pectinases. These lesions hinder carbon uptake and photosynthesis by decreasing the functional leaf area [62].

In the present study, an association of AM fungal species was engaged to evaluate its effectiveness as a pre-inoculation strategy for enhancing the resistance of *L. sativa* L. against *A. alternata* RaSh3. While this approach reflects practical agricultural applications, where mixed AM inocula are commonly used, it does not permit the identification of specific synergistic or antagonistic interactions among fungal partners. Interestingly, AM fungal inoculum applied as a mixed culture decreased the leaf spot disease of *A. alternata* RaSh3-infected lettuce plants as compared to non-mycorrhizal-infected ones. This behavior has been described by Fritz et al. [65], who found that *G. intraradices* decreased necrosis and chlorosis. According to Wang et al. [25], inoculating tomato plants with *R. irregularis* considerably reduced the Fusarium wilt disease index. Also, *G. clarum* application effectively controls *A. solani* on tomatoes [24]. The disease index and incidence of *Ralstonia solanacearum* inoculated with *G. rhizogenes* and *G. mosseae* were 9.7% and 49.8% lower, respectively, than the control group that skipped the AM fungal inoculation [66].

Furthermore, the biocontrol effects of *Gigaspora* spp., *Paraglomus* spp., *Glomus* spp., *Rhizophagus* spp., and *Funneliformis* spp. on a number of fungal infections were compiled in a recent review by Abarca et al. [67]. Altering the morphology or anatomical structure of plant roots, enhancing the rhizosphere environment, improving plant nutrition, competing with pathogens for photosynthetic products and infection space, triggering disease resistance defense mechanisms in plants, and controlling the production of secondary metabolites in host plants [68, 69] are all mechanisms involved in AM fungal improvement of plant disease resistance. Indeed, the host plant’s root system can grow, thicken, and branch more when AM fungal symbiosis occurs. It can also speed up the lignification of the cell wall, thicken the root tip epidermis, and increase the number of cell layers. Additionally, it can alter the morphological structure of the roots, which effectively slows down the process of pathogen infection [67, 70]. Moreover, on the surface of the inner and outer hyphae of the root, AM fungi can create chemicals, including phytochemicals, calloses, alkaloids, and phenols, which help plant withstand unfavorable conditions [71]. Nevertheless, the observed improvements in plant performance and disease suppression may be attributed to the functional complementarity of the AM fungal community, which can enhance colonization efficiency and widen the physiological benefits conferred to the host plant. Future research should aim to employ single species to elucidate species-specific roles and interaction dynamics.

In this study, AM fungi that can increase trigonella plant growth and Cr-stress tolerance [30] were chosen for additional research on their capacity to trigger defense mechanisms in plants against the *A. alternata* RaSh3 pathogen infection. Our findings on lettuce root colonization are consistent with Miozzi et al. [72], who found that when compared to mycorrhizal non-infected plants, the percentage of arbuscules throughout the entire root system and the intensity of mycorrhization in tomato plants inoculated with the AM fungal colonization caused by the cucumber mosaic virus showed a slight increase. Conflictingly, Klinsukon et al. [73] stated that plant roots treated with both AM and a pathogen (*Cylindrocladium quinqueseptatum* KKU-H4-B12), the colonization (*G. margarita*) decreased, resulting in leaf blight disease in eucalyptus seedlings.

Although the beneficial effects of AM fungi on plant growth and disease resistance have been widely reported, in the present study, we specifically investigated the importance of AM early symbiotic establishment in *L. sativa* L. plant defense responses prior to the *A. alternata* RaSh3 pathogen challenge, thereby offering a proactive disease management strategy rather than a reactive one. Plant growth and development are significantly hampered by the necrotrophic fungus *A. alternata* due to direct tissue destruction, physiological process disruption, and diversion of metabolic resources. Our study’s findings about the detrimental effects of *A. alternata* RaSh3 infection on lettuce growth response were consistent with those of Zhang et al. [9] and Abdelhameed et al. [62] who found that *A. alternata* had a negative effect on potato and pepper plants, where infection was associated with a marked decrease in SH, RL, Fwt, and Dwt. *A. alternata* is the source of the highly devastating Alternaria blight disease; it results in leaf and stem blight, which defoliates leaves and drastically lowers their growth and quality [74]. One may argue that pathogenic fungi create a variety of toxins that disrupt the regular physiology of plants by facilitating the nutrient escape from injured tissues [75], which finally results in an overall reduction in plant biomass [76]. The decline in lettuce’s Fwt could be attributed to the activity of phenolic chemicals from the fungal infection-induced depolymerization of the cell wall’s lignin. Furthermore, the fungi’s toxins reduce the RFwt by affecting K^+^ absorption and stomata function, which results in uncontrollable transpiration and excessive water loss; this increases membrane permeability and causes plant wilting [23, 77].

Our findings were consistent with earlier findings that, when compared to infected plants alone, AM fungal colonization improved plant growth parameters and reduced early blight disease in tomatoes caused by *A. solani* [78], white rot disease in garlic plants caused by *Sclerotium cepivorum* [79], and alfalfa leaf spots caused by *Phoma medicaginis* [80]. In the study of Pu et al. [81], Fusarium wilt brought on by *F. oxysporum* was much reduced when *Salvia miltiorrhiza* was pre-inoculated with *G. versiforme* due to an increase in macro- and micronutrient uptake from the soil that is unavailable to roots through the extra-radical mycelium network of AM fungi, particularly of P and Zn [30, 82, 83], hence reducing the metabolic drain caused by pathogens and ultimately increasing the buildup of biomass [84].

According to Serrano et al. [85], photosynthesis not only supplies nutrients for plants but also generates defense-related compounds to fend against infections. In comparison to control plants, *A. alternata* infection reduced the levels of carotenoid, Chl a, Chl b, and Chl (a + b) of lettuce plants. Likewise, when *A. alternata* and *S. cepivorum* were introduced to pepper and garlic plants, the amount of pigments decreased in comparison to the plants that were not infected [62, 79]. The disintegration of the plastid membrane during infection may be the cause of this decrease [86]. Gómez-Gallego et al. [87] claimed that hyphal growth can obstruct the stomatal opening and hinder photosynthesis, which in turn can induce decreases in stomatal conductance during an attack by *A. alternata*. Additionally, toxins produced by *A. alternata* can reduce the photosynthetic area of leaves and impair the host’s defensive response [88]. Additionally, the pathogen-induced necrotic patches on infected leaves were more noticeable, suggesting that the pathogen caused the photosynthetic machinery to malfunction, which in turn led to cell death [89].

The stabilization of chloroplast membranes via AM-induced antioxidant activity further lessens photo-oxidative damage, ensuring carbon assimilation. A comparable finding about the impact of AM colonization on lettuce plant pigment fractions was made by Rashad et al. [79] and Pu et al. [81]. According to Abdelhameed and Metwally [84] and Evelin et al. [90], AM plants use extra-radical mycorrhizal hyphal structure to improve Mg absorption and retain more Zn, which are essential for photosynthesis and increase the chlorophyll concentration. According to Fritz et al. [65] and Dehne [91] the systemic influence of AM fungi may be linked to improved mycorrhizal plant growth, nutrition, and physiological activity, which in turn may result in higher assimilate levels. Given that both symbiotic and pathogenic organisms rely on host photosynthates for growth, competition for these carbon molecules (assimilates) may have contributed to a reduction in pathogen development in mycorrhizal plants. In addition to chlorophyll, the mycorrhizal symbiosis considerably raised the carotenoid content of infected lettuce plants. All of these findings point to the possibility that increased photosynthesis brought about by AM fungal inoculation may enhance lettuce plants’ resistance to disease.

One of the quickest reactions to a microbial infection that plants are known to exhibit is the oxidative burst, or the rapid generation of ROS. Plant redox equilibrium is significantly disrupted by Alternaria infection, as evidenced by the buildup of H_2_O_2_ and MDA, two oxidative stress markers [92]. H_2_O_2_, a ROS, serves as a dual-edged sword in plant-pathogen interactions. While moderate H_2_O_2_ levels act as signaling molecules to activate defense pathways, *A. alternata* induces excessive H_2_O_2_ production in lettuce plants. Its elevated concentration in cellular systems positively correlates with the oxidative changes affecting MDA content, which is the marker for lipid peroxidation released from cellular membranes and is formed by the reaction of ROS (H_2_O_2_ or/and superoxide radical) with lipid molecules [93]. This result is consistent with Philip et al. [63], who exhibited raised levels of MDA and H_2_O_2_ in infected tomato plants. El-Khallal [94] conveyed that increased buildup of H_2_O_2_ may lead to accelerated senescence and decreased photosynthetic rate in infected tomato plants. Besides, Lubaina and Murugan [92] have reported that *A. sesami* infection increases the amount of lipid peroxidation in the pathogen-inoculated *Sesamum orientale* leaf samples with amplified MDA content.

AM fungi mitigate the oxidative stress in *A. alternata*-infected plants by modulating ROS homeostasis and suppressing lipid peroxidation. Recent studies [33, 95] validate that AM colonization lessens pathogen-induced H_2_O_2_ and MDA increment through enhanced antioxidant activity and improved nutrient status. Remarkably, after AM treatment, the MDA and H_2_O_2_ levels of lettuce plants were much lower than those of non-colonized plants. This could be due to AM fungal colonization causing AM plants to grow more than non-colonized ones that may alleviate the damage caused by pathogens, lowering oxidative stress marker concentration [83], stabilizing cell membrane integrity, and dropping electrolyte leakage.

Infected plants prioritize defense over growth, redirecting carbon and nitrogen toward synthesizing phenolic compounds, flavonoids, and pathogenesis-related proteins. For example, lignin deposition in cell walls reduces pathogen spread but limits cell expansion, contributing to stunted growth [96]. This trade-off between defense and growth is evident in infected lettuce in our study, where a reduction in soluble carbohydrates occurs due to their preferential use in defense metabolite synthesis. Consistent with our findings of high protein content in AM-colonized lettuce plants, Pu et al. [81] demonstrated that inoculation with AM fungi (*G. versiforme*) increased the protein content of *S. miltiorrhiza* infected with *F. oxysporum*. Under stressful conditions and with AM inoculation, Abdelhameed and Metwally [30] confirmed the increase in osmolytes like protein, proline, and carbohydrates, which build up as antioxidants that counteract dangerous ROS. Moreover, these osmolytes protect against denaturation of proteins and disintegration of cell membranes. They also make enzymatic proteins structurally stable, which keeps them functional and reduces the amount of water needed for active metabolism, extending plant longevity [97]. Through osmotic adjustment and reflecting N availability for plant growth and development, soluble proteins also shield plants from stress [98, 99]. Therefore, lettuce plants treated with AM fungi provide protection against *A. alternata* due to the accumulation of proline, carbohydrates, and proteins.

Our findings of increasing PAL activity are in line with Rashad et al. [79] and Eke et al. [100], who demonstrated that AM fungal application led to the greatest increase in the activity of the defense-related enzyme, PAL, in common bean and garlic plants against Fusarium root rot and white rot disease. The PAL activity of mycorrhizal and pathogen-infected *S. miltiorrhiza* was significantly increased by 39% compared with that of the control [81]. A study by Wang and Chen [33] demonstrated how AM fungi can increase systemic resistance by activating PAL that is involved in the phenylpropanoid pathway, which produces a variety of secondary metabolites [62, 101]. These secondary metabolites include lignin (a quickly deposited physical barrier) and several antimicrobial substances (phenolic and flavonoids), all of which are linked to plant pathogen tolerance [102]. Phenolics can either act as soluble antimicrobials or cross-link into cell walls with proteins and callose [103]. Our findings are supported by Philip et al. [63], who found that large amounts of phenolic content accumulated in tomato plants infected with *A. alternata*. Increased phenolic levels might offer sufficient substrate for peroxidase-catalyzed oxidative reactions, leading to lignification and rendering plant cells unsuitable for the growth of further pathogens [104].

The mechanism of triggering host cells for the production of some fungi-toxic phenolic compounds as a result of AM colonization was established by the results attained in the current study, which correspond with Rashad et al. [79] and Eke et al. [100], demonstrating the firming of the plant immune system against the pathogen. El-Sharkawy et al. [105]. discovered that phenolic chemicals were more abundant in rust-infected wheat plants inoculated with AM fungi than in non-mycorrhizal ones. Additionally, tomatoes and sunflowers produce flavonoids in response to AM fungal colonization, which protects them from invasive infections (68; 79). Additionally, the increased phenolic metabolism is assumed to be the cause of the improved cell wall lignification, which inhibits pathogen penetration [106]. Wianowska et al. [107] demonstrated that phenols could hinder mycelial growth of *A. alternata*, among other pathogens. This effect could be attributable to the capability of these compounds to obstruct fungal spore germination [108]. It has been verified that methyl *p*-coumarate, a phenolic compound, has antifungal activity against *A. alternata* by inhibiting mycelial growth and spore germination [109].

Our results of the effect of *A. alternata* RaSh3 on CAT, POD, and APX of lettuce plants are in line with Philip et al. [63], who showed the greatest CAT, POD, and polyphenol oxidase activity in tomato plants infected with *A. alternata* compared to the control plants. Debona et al. [110] examined the pathogen-induced biochemical alterations in wheat and found higher levels of CAT activity, confirming its function in promoting disease resistance. The elimination of extra H_2_O_2_ generated was shown by the increased CAT activity [111]. One antioxidative pathway that removes the excessive H_2_O_2_ is an ascorbate-glutathione cycle [112], which embraces a series of redox reactions involving enzymes like APX, dehydroascorbate reductase, and monodehydroascorbate reductase [56]. APX is responsible for eradicating extreme H_2_O_2_ from chloroplasts, peroxisomes, and mitochondria [113]. Furthermore, experimental data from Jackson et al. [114] and Jin et al. [115] studies linked POD to plant defense responses to pathogen attacks because it is crucial for the cross-linking of cell wall components, which fortifies cell walls to prevent pathogen invasion. Obviously, AM fungi activate systemic resistance to pathogens [83, 116], which involves activation of defense-related enzymes such as PAL and POX that are associated with the production of phenolic and structural barriers [33, 79, 94], and all of these make possible the activation of a powerful defense system in lettuce against attack of *A. alternata*.

Although the present study did not include molecular-level analyses of defense-related genes such as *LOX*, *PDF1.2*, or *PR* proteins, the observed improvements in disease resistance and biochemical responses of *L. sativa* L. following AM fungal pre-inoculation can be interpreted within the induced systemic resistance (ISR) and are indicative of a primed physiological state. The significant upregulation of antioxidant enzymes such as POD, PPO, and APX, together with reduced disease severity, suggests that AM colonization primes the plant’s defense system, enabling an effective response upon *A. alternata* RaSh3 infection. This priming effect is likely associated with enhanced regulation of ROS and activation of downstream defense pathways. Nevertheless, the lack of transcriptomic or RT-qPCR validation limits definitive conclusions regarding the specific signaling pathways involved. Therefore, future studies integrating gene expression and phytohormonal profiling are recommended to expand the proposed mechanistic model of AM-mediated resistance in lettuce, validate the universality of these mechanisms, and enhance the broader applicability of AM fungi as biocontrol agents in sustainable agriculture.

In this study, *A. alternata* RaSh3 infection can diminish ACP and ALP activities in lettuce plants, which may lead to impaired P mobilization. The reduction in phosphatase activity may be due to tissue damage, enzyme degradation, or pathogen-induced suppression of these enzymes. On the other hand, mycorrhizal colonization induces systemic changes in plant metabolism, leading to elevated phosphatase expression in both roots and associated fungal structures. Mycorrhizal colonization significantly influences the activity of both phosphatases in plants [30, 97]. These enzymes play a vital role in P acquisition by catalyzing the hydrolysis of organic phosphorus compounds into inorganic forms that can be absorbed by plant roots. ACP activity generally upsurges more than ALP activity, as acid phosphatases are dominant in acidic and neutral soils, where organic phosphorus needs to be mineralized into inorganic phosphate for plant uptake. ALP also contributes, especially in calcareous (alkaline) soils, but to a lesser extent compared to ACP [117].

## Conclusions

In the agricultural industry, alternative, environmentally acceptable bioagents are needed to combat plant diseases. This study is the first to describe the use of AM fungi to improve lettuce plant growth and assist them in fending off *A. alternata*-caused leaf spot disease. *A. alternata* RaSh3 displayed a highly significant decrease in all growth traits of shoot, root, and leaf and chlorophyll contents, accompanied by a boost in oxidative stress markers. Application of AM fungi activated systemic resistance to the *A. alternata* pathogen by activation of osmolytes, phosphatases, antioxidant, and defense-related enzymes like PAL, CAT, APX, and POD, in addition to total phenolic and flavonoids. All of these lead to the activation of the power defense system in lettuce against the attack of *A. alternata* RaSh3. By integrating analyses of disease severity, plant growth performance, and biochemical defense markers, our work provides new insights into the mechanistic basis of AM-induced resistance under pre-colonization conditions. This contributes to advancing current understanding of AM fungi as sustainable bio-agents and as an environmentally benign substitute for chemical fungicides, particularly in leafy vegetable crops where foliar diseases significantly limit productivity. To assess their effectiveness, survivability, and microbial interactions with the soil microbiome, we recommend future research on the use of the AM fungi in open field settings.

## Data Availability

All data generated or analyzed during this study are included in this published article.

## Abbreviations

ACP: Acid phosphatases
ALP: Alkaline phosphatases
A%: Arbuscules abundance
AM: Arbuscular mycorrhiza
APX: Ascorbate peroxidase
CAT: Catalase
DI: Disease incidence
DR: Disease reduction
DS: Disease severity
F%: Mycorrhization frequency
GAE: Gallic acid equivalent
MDA: Malondialdehyde
MD: Mycorrhizal dependency
M%: Mycorrhization intensity
PAL: Phenylalanine ammonia-lyase
POX: Peroxidase
QE: Quercetin equivalent
RL: Root length
ROS: Reactive oxygen species
RFwt: Root fresh weight
SDwt: Shoot dry weight
SH: Shoot height
TAC: Total antioxidant capacity
TCA: Trichloroacetic acid
OD: Optical density
